# 

**Published:** 1884-10-20

**Authors:** 


### TABLE OF CONTENTS.

ORIGINAL AND SELECTED ARTICLES:

Silver Wire Sutures in Wounds of the Intestines, by J. McF. Gaston, M.D, Atlanta, 361. French Theories on Cholera, 364 Quassia as a Cure for Cholera, 367. The Greely Expedition and Anthropophagy, 368. Some Notes on Medical Jurisprudence, 370. Treatment of Piles by Injections, 372. Pilocarpine in the Treatment of Ascites Caused by Disease of the Liver, 374. Celloidin as an Embedding Mass, 375. Bichloride of Mercury, 376.

ABSTRACTS AND GLEANINGS:

Vaccine Virus Efficacious Without the Production of Cutaneous Manifestations; On Opening and Draininge of Abscess Cavities in the Brain, 377. The Castration of Women, 378. Hot Water and Inflamed Mucous Surfaces, 379. Glycerine as a Remedy in Indigestion; Tyndall's Theory of Explaining the Immunity Obtained against a Second Attack of Contagious Disease, 380. The Number Seven, 381. Bromidia and Morphia in Delirium Tremens, 382. Glycerite of Tar; Delirium Tremens, 383. Ergot in Dysmenorrhœa; To Prevent Bed Sores; Keeping Cider Without Boiling, 384. Male or Female Offspring at Will; Universal Erysipelas in a Chi d Aged Ten Months, with Recovery, 385. Vomiting of Pregnancy, 386. Treatment of Consumption; Cholera Injection; Cocoa, 387. Copper as an Antidote to Cholera; Deep Breathing; Post-Nasal Catarrh, 388. Conium in Malarial Disease; The Bromides as a Cause of Insanity; Witch Hazel in Menorrhagia, 389. A New Disease; Treatment of Ingrowing Toe Nail; The Globus Hystericus; Cure of Hydrocele; Cholera Preventives, 390.

SCIENTIFIC ITEMS:

The Banyan Tree; A Paper Door; A Curious Calculation, 391. Electricity in Dental Practice; Electric Ballet, 392.

PRACTICAL NOTES AND FORMULÆ.

Thielemann's Cholera Mixture; Esmarch's Painless Caustic, 393. Formula for S. S. S.; Thompson's Eye Water; Pulv. Anti-Chol. Infanti; For Piles, 394. Emmenagogue; Red color for Show Globes; Hill's Balsam of Honey and Tar, 395.

EDITORIALS AND MISCELLANEOUS.

Editorial Notices; Opening Exercises of the SOUTHERN MEDICAL COLLEGE, 396 In Memoriam; Mortality in Atlanta; Gov. Cleveland and the Homœopaths, 397. Proceedings of the Chicago Medical Society; Books and Pamphlets Received, 398. Receipted; Special Notices, 400.

